# Transactions of the Chicago Society of Physicians and Surgeons

**Published:** 1875-05-01

**Authors:** Ralph E. Starkweather


### TRANSACTIONS OF THE CHICAGO SOCIETY OF PHYSICIANS AND SURGEONS.

REGULAR MEETING, APRIL 26TH, 1875.

*Reported by Ralph E. Starkweather, M.D.*

THE President, Dr. John Bartlett, in the chair.

Dr. Plym S. Hayes read a carefully prepared paper upon "Experimental Observations on Salicylic Acid," of which the following is a brief summary:

After stating and reviewing the opinions of Dr. Squibb concerning this acid, and quoting from Dr. Dalton in regard to the action of the digestive fluids, Dr. Hayes criticizes the expression catalysis, and states that in the old chemistries catalysis occupied a place of great importance, and was the pack-horse on whose back was strapped many of the inexplicable phenomena connected with chemistry. The new text-books generally ignore this expression. Fermentation, which has in a measure supplanted the term catalysis, refers, in its most restricted sense, only to that action, the cause of which is almost entirely due to living organisms. Dr. Squibb calls this class of ferments "*vital*," of which that produced by yeast, and many changes which occur in putrefaction, are examples. That chemical action in which living organisms have no influence, but the metamorphosis is caused by the action of some physical agent, or of some definite chemical compound elaborated by an organism—some organic substance—the same authority calls "*chemical*," being those which occur independent of vitality, as the production of volatile oil in mustard and bitter almonds, the effects of diastase, and similar changes. Dr. Hayes details two different series of experiments, in which he used some of Dr. Squibb's unbleached salicylic acid. The first series was instituted to determine whether the acid had the power of preventing the formation of glucose when saliva had been added to starch which contained a portion of acid. The object of the second series was to ascertain whether the acid arrested or retarded in any degree the action of the gastric juice, or its active principle, pepsin, on coagulated albumen.

Next follows the account of the individual experiments which had been conducted with scholarly and scientific precision; the results attained, and the chemical agents and tests employed, being in turn re-tested and verified by other methods and processes.

The following are the conclusions drawn by Dr. Hayes: First, that salicylic acid retards digestion in a marked degree, when performed artificially. Second, that it slowly coagulates albumen.

Dr. Hayes thought that we might look for different effects in the administering of the acid, according as it may be exhibited by the mouth or by topical application to an abrasion, provided the statements of Prof. Kolbe and Dr. Squibb are each correct. Prof. Kolbe says that given internally, no trace of the acid is discoverable in the urine or fæces. Dr. Squibb states that when the acid is applied to wounds, it appears immediately in the urine (quoting from Thiersch, *Pharm. Centrahalle*).

The paper was discussed by Drs. Bartlett and Hyde.

CANCER OF THE STOMACH.

Dr. Etheridge gave the clinical history and treatment of this case, assisted by Dr. I. N. Danforth, who had made the post-mortem examination, and exhibited to the Society, by means of the microscope, numerous pathological specimens taken from this case. The paper is reserved for publication.

The discussion which followed called forth among other facts the noticeable one that the patient, with other members of her family, had thirty years previously partaken of a broiled chicken which had been kept in a new refrigerator. The day following the meal the entire family were prostrated by acute arsenical poisoning. From that time forward the patient had been subject in summer to bilious colic, and had been obliged to refrain from certain articles of food.

Dr. Danforth thought that there had at first been simple inflammation of the pyloric opening of the stomach, and that this tissue later in life became invaded by true cancer cells. Epithelial cells, provoked from their regular growth, may become atypical, and so, after years of constant irritation, become malignant—or epigrammatically, *The lawlessness of growth is the essence of malignancy*, speaking histologically.

QUINIA AND ITS ALKALOIDS.

Mr. Albert E. Ebert, late president of the American Pharmaceutical Association, addressed the Society briefly as follows:

There are a number of alkaloids in the Peruvian bark, quinia being the best known and most largely employed. There are besides (1) quinidia, (2) cinchonidia, and (3) cinchonia. The cinchonia in the red bark is nearly as abundant as quinia.

There has been an immense amount of these "cheaper" alkaloids made while manufacturing quinia, and there having been no demand for these other alkaloids, they have been stored away and their cost has been added to that of quinia.

The cheaper alkaloids are said by reputable members of the medical profession to have the same or nearly the same therapeutical effect as quinine. The manufacturers have made efforts to introduce these bi-products (the cheaper alkaloids) to the profession, and rid their stock of these alkaloids that have accumulated for years while making quinia.

Sweet quinine was then spoken of, and the history of the exposure of the fraud, and expulsion of certain parties from the American Pharmaceutical Association.

Cincho-quinine was next referred to. If the testimonials from physicians in regard to this nostrum are true, then cinchonia sulphate has a large medicinal virtue, for none can be attributed to the quinia it is said to contain.

Mr. Wentzel, of California, a competent and conscientious chemist, has examined this nostrum, and found that it contained cinchonia only.

In 1873, Mr. Ebert had himself examined seven different original samples, microscopically and chemically, and did not find a trace of quinia in any of them.

In the wholesale market not one of the original packages made before 1874 can be had; they have either been used or bought up.

The samples now sold do not contain the alkaloid quinia but the sulphate salt of quinia, and this only in the *six-tenths of one per cent.* (6–10 of 1 per cent.)

The success of these alkaloids goes to show that in many respects they are as useful in therapeusis as quinia sulphate, and as they cost only twenty (20) cents per ounce, are much cheaper. If extensively used the price of quinine would be reduced, and the rapid destruction from the forests of these precious trees would be much lessened.

### QUININE BRUTE.

In reply to a question, Mr. Ebert said that the article generally dispensed under the term quinine brute is chinodine, or the precipitated extract of cinchona bark, such as is found in the market under the name of "Wetherill's" or "Ellis'" extract of calisaya bark.

The process is published in Parrish's Practical Pharmacy, fourth edition, p. 665.

Trousseau, in his work on Therapeutics, says that quinine brute is a combination of all the cinchona alkaloids in the impure state.

Chinoidine is the residue after the crystallizable principles have been extracted from the mother liquid, evaporated down to a solid, extractive consistency. This preparation has the same relation to the crystallizable alkaloids that molasses has to sugar.

Dr. Etheridge had found that children and others could take quinine agreeably, if given in the syrp. glycyrrhiza. In one instance, a gentleman unable to take quinine was effectually relieved by twenty-grain doses of quinine brute.

Dr. Starkweather referred to an article on intermittent fever by Bouchut, which considered that quinine brute was the most useful remedy to prescribe for children. It is less soluble and less bitter, and more easily taken by children. It should be exhibited just after the fever, in the dose of three to six grains daily. This is nearly double the dose of quinine recommended to be given to children, and adds that it should be given by enema.

The Society, after transacting considerable routine business, adjourned.

---

OATMEAL, BONE AND MUSCLE.—Professor Forbes, of Edinburgh, during some twenty years, measured the breadth and height, and also tested the strength of the arms and loins of the students in the University—a very numerous class, and of various nationalities, drawn to Edinburgh by the fame of his teaching. He found that in height, breadth of chest and shoulders, and strength of arms and loins, the Belgians were at the bottom of the list; a little above them the French; very much higher the English; and highest of all, the Scotch and Scotch-Irish, from Ulster, who, like the natives of Scotland, are fed in their early years with at least one meal a day of good milk and good oatmeal porridge. —*Pacific Medical and Surgical Journal.*

